# Endoscopic retrograde appendicitis therapy: current and the future

**DOI:** 10.1093/gastro/goae037

**Published:** 2024-04-27

**Authors:** Dan Liu, Jiyu Zhang, Bingrong Liu

**Affiliations:** Department of Gastroenterology, The First Affiliated Hospital of Zhengzhou University, Zhengzhou, Henan, P. R. China; Department of Gastroenterology, The First Affiliated Hospital of Zhengzhou University, Zhengzhou, Henan, P. R. China; Department of Gastroenterology, The First Affiliated Hospital of Zhengzhou University, Zhengzhou, Henan, P. R. China

**Keywords:** endoscopic retrograde appendicitis therapy, appendicitis, super minimally invasive therapy, appendicitis treatment

## Abstract

This article presents an overview of endoscopic retrograde appendicitis therapy (ERAT), an innovative and minimally invasive treatment for appendicitis with an appendix-preserving manner. Since its initial application in 2009, ERAT has gained significant popularity in China, due to its rapid recovery and minimal risk of complications. The ERAT procedures comprise several steps, including appendiceal orifice access and intubation, appendiceal lumen imaging, decompression and irrigation, fecalith removal, and stenting. ERAT has been used in various forms of complicated appendicitis, such as in pregnant women and children, with continuous improvements in both technique and safety. It has the potential to become the preferred diagnostic and treatment method for appendicitis. Until 2023, over 10,000 ERAT procedures have been successfully conducted in China, and the technique has gained more attention worldwide. However, challenges remain, including training, standardization of ERAT practice, research and technology improvement, enhancing public awareness, and fostering international collaboration. In summary, ERAT can be the standard treatment for appendicitis treatment, which represents a paradigm shift in the conventional clinical practice.

## Introduction

Appendicitis is one of the most common abdominal emergencies, mostly affecting children and young adults, with a lifelong risk of 7%–8% for each individual [1, 2]. The etiology of appendicitis usually relates to appendiceal luminal obstruction due to fecaliths, lymphoid hyperplasia, infectious agents such as parasites, foreign bodies, and tumors. Appendiceal obstruction leads to excessive bacterial growth, inflammation of the appendix wall, and increased intra-appendiceal luminal pressure [3, 4]. Once the inflamed appendix is left unmanaged, gangrene, perforation, abscess or sepsis may occur. Appendectomy and conservative antibiotics had been considered the treatment of first choice for uncomplicated appendicitis [5–7].

Inspired by endoscopic retrograde cholangiopancreatography for the management of acute obstructive suppurative cholangitis, Bingrong Liu performed the first case of endoscopic retrograde appendicitis therapy (ERAT) in 2009 and reported it at the American Digestive Diseases Week conference in 2011. The ERAT technique was first published in *Gastrointestinal Endoscopy* in 2012 [8]. ERAT is a novel, super minimally invasive endoscopic technique for appendicitis treatment in an appendix-preserving manner [9]. It has technical feasibility, due to easy access to the appendix using colonoscopy. Appendiceal intubation, fecalith removal, and appendiceal lumen flushing with normal saline are the main steps in the ERAT procedure. The advantages of ERAT include no skin wound, organ preservation, immediate relief of abdominal pain, no postoperative pain, early food intake, rapid recovery, fewer postoperative complications, and shorter or no-need hospitalization.

ERAT is capable of relieving appendiceal obstruction and achieving both causative and curative treatment for appendicitis. After more than ten years of clinical practice, ERAT has been widely used in China, with favorable outcomes, including its rapid recovery, almost non-invasive nature, lower risk of adverse events and acceptable recurrence rate [10–13]. This review encompasses the technical essentials, current status and future perspectives of ERAT, hoping to provide reference and assistance for the further development and global promotion of ERAT technique.

## Procedural steps of ERAT

### Bowel preparation

Similar to colonoscopy, adequate bowel preparation is essential for the ERAT procedure. In emergency cases, a rectal enema can be utilized as a complementary to oral bowel preparation. In addition, an intracolonic cleansing machine can also be used for rapid bowel preparation.

### Appendiceal intubation

**Figure 1.**
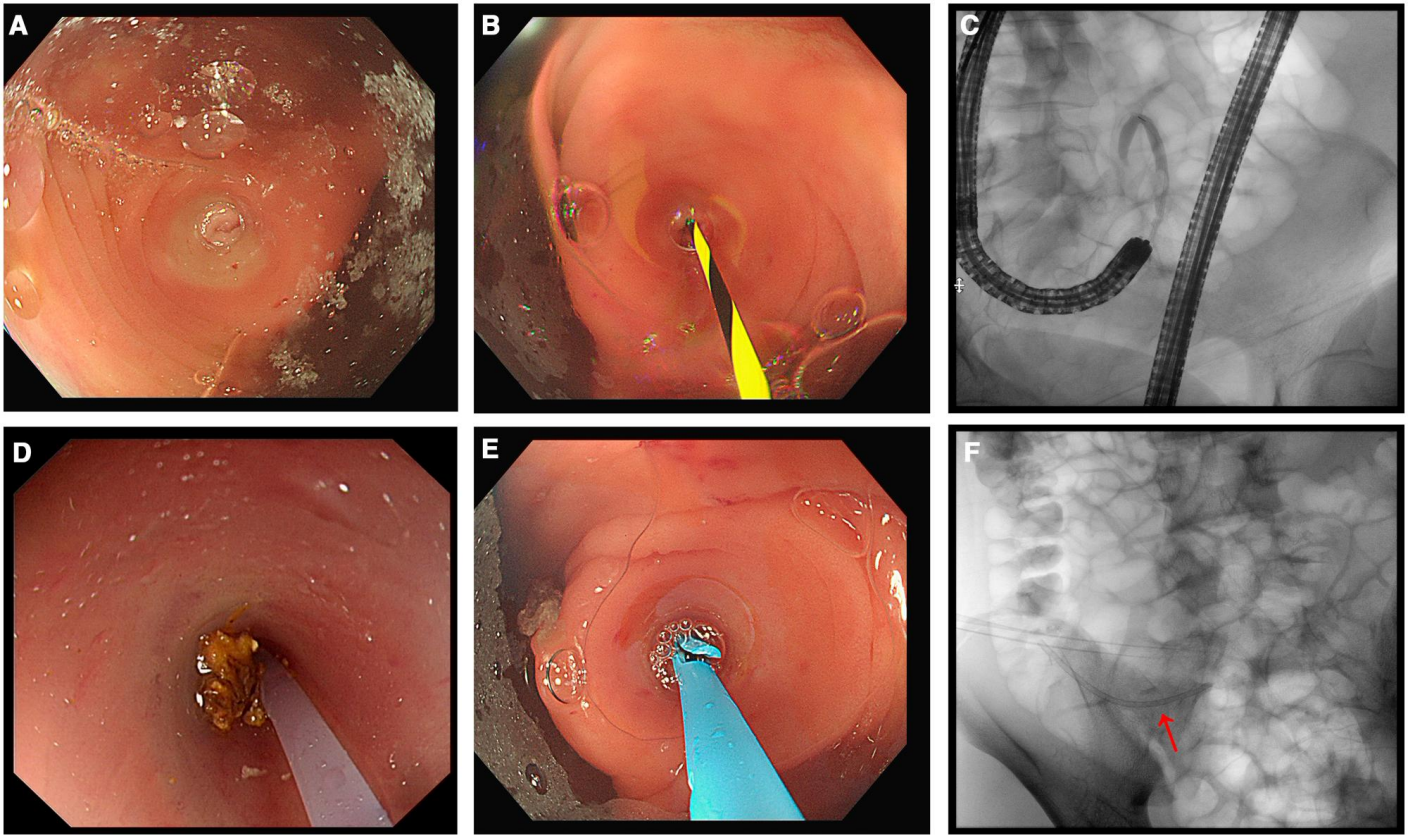
Endoscopic retrograde appendicitis therapy procedure. (A) Finding and exposing the orifice of the appendix. (B) Guidewire-guided intubation. (C) Appendicography. (D) Flushing out pus and fecalith. (E, F) Stent placement.

The most classic and commonly used operating method of Endoscopic Retrograde Appendicography remains to be under X-ray guidance. A transparent cap with a tapered shape is recommended to use on the tip of the colonoscope, in order to push aside the Gerlach valve and identify the orifice of the appendix (Figure 1A, Video 1). The lumen of the appendix was intubated using the guidewire-catheter technique under X-ray guidance (Figure 1B). Abdominal ultrasound is also an applicable and effective guidance in some experienced units, especially suitable for pregnant women and children.

### Appendiceal lumen imaging

Under X-ray guidance, the lumen of the appendix was imaged with a diluted water-soluble contrast agent to evaluate the morphology of the appendiceal lumen (Figure 1C). The presence of appendiceal dilatation, stenosis, filling defects, or contrast leakage is the main characteristic imaging to obtain the optimal diagnosis of appendicitis, appendiceal fecalith, or appendiceal perforation. Recently, a peroral cholangioscopy system (SpyGlass™ DS II Direct Visualization System, Boston Scientific Corporation, MA, USA; and Single-use Video Pancreaticobiliary Scope, eyeMAX, Jiangsu, P. R. China) was applied in selected patients for ERAT in P. R. China.

### Appendiceal decompression and irrigation

After successful intubation, purulent fluid inside the appendiceal cavity can rapidly flow out to release the internal pressure of the appendix, thus alleviating appendicitis referral symptoms. Some fecaliths can be flushed out of the appendiceal cavity (Figure 1D). Operators should pay attention to the color of the flushing fluid from the appendix to ensure the clearance of the appendiceal lumen. After thorough irrigation, the morphologic appearance of the appendix will be re-evaluated by contrast injection under X-ray guidance.

### Appendiceal stone removal

If the appendiceal fecalith cannot be expelled by appendiceal irrigation, retrieval baskets or balloons can be used under X-ray or abdominal ultrasound guidance. The peroral cholangioscopy system can also be used together with laser lithotripsy for lithotripsy and removal of the obstructive fecalith [14].

### Appendiceal stenting

When suppurative appendicitis and luminal stenosis were diagnosed, a 7–8.5-Fr plastic stent (5–7 cm in length) was recommended (Figure 1E and F). Stenting aims to drain residual pus and support the narrow lumen to reduce the luminal pressure of the appendix. In clinical practice, an appendiceal stent is usually retrieved in about 4 weeks or more, after ensuring it is *in situ* by abdominal radiography or CT scan. Actually, some stents can discharge from the appendiceal cavity spontaneously.

## Recent progression in ERAT

### Limitation of post-onset time

The key to treating appendicitis lies in early diagnosis and evaluation. Although traditional methods of diagnosing appendicitis rely on combining symptoms, physical examination with imaging results, severe complications such as appendiceal perforation may occur due to missing the optimal interventional window. Importantly, ERAT can be used as a treatment method, but also as a diagnostic strategy.

### Expansion of ERAT indications and contraindications

ERAT was indicated for acute uncomplicated appendicitis at the beginning. With the increasing use of ERAT technology in clinical practice, some cases of complicated appendicitis were treated using ERAT successfully and achieved satisfactory outcomes. More and more evidence shows that even selected complicated appendicitis can be an indication of ERAT. The contraindications to ERAT are patients who are unable to undergo routine colonoscopy, such as patients with severe cardiopulmonary dysfunction, inadequate bowel preparation, etc.

#### Acute complicated appendicitis

Complicated appendicitis refers to appendicitis with gangrene of the appendix, perforation, or periappendiceal abscess, which usually requires urgent consideration of appendectomy. In 2021, Song *et al.* [15] reported a case of complicated appendicitis with periappendiceal abscess in a critical patient who was unsuitable for appendectomy; the result of this patient proved to be effective and curative. Zhang *et al.* [16] compared laparoscopic appendectomy with ERAT for complicated appendicitis and demonstrated that the ERAT group effectively relieved symptoms, and the operation time, ambulation time, and hospital stay were shorter than the laparoscopic appendectomy group.

#### Appendiceal mucocele

The previous appendectomy is the only choice for appendiceal mucocele. ERAT demonstrates superiority in relieving the etiology of appendiceal dilatation via stenting and appendiceal dilation. Li *et al.* [17] used ERAT to treat 8 cases of appendiceal mucocele, and re-examination colonoscopy showed no appendix stenosis and residual lesions in 8 patients, demonstrating that ERAT is safe, effective, and feasible in the treatment of appendiceal mucocele.

#### Stump appendicitis

Stump appendicitis is a rare complication after a primary appendectomy; non-specific clinical presentation usually makes the diagnosis of stump appendicitis challenging. A longer stump has been thought to present a risk for fecalith obstruction, which had been considered the most common cause of stump appendicitis. Stump appendicitis has been managed with ERAT, with intubation and fecalith removal, which proves to be a safe and effective therapy [18].

#### Application of ERAT in special clinical condition

##### Application of ERAT in pregnant women

Acute appendicitis in pregnancy represents a unique diagnostic and therapeutic challenge. A delay in diagnosis and treatment can lead to adverse outcomes for both the mother and fetus. Due to the reluctance to use radiological applications for fear of harming the fetus, ERAT is usually performed and orientated under peroral cholangioscopy system or abdominal ultrasound guidance. ERAT has been successfully performed for a woman in the first trimester of pregnancy and achieved clinical success following an uneventful delivery [19].

##### Application of ERAT in children

Appendicitis represents the most common abdominal surgical emergency in children. The appendix plays a prime role in immune defense and serves as a reservoir for beneficial intestinal flora. As a result, appendix-preserving is extremely important in children. ERAT has proven to be a feasible and desirable option for pediatric appendicitis [20]. Abdominal ultrasound and cholangioscope as guidance during ERAT are strongly recommended to avoid radiological damage.

### The development of orientation during ERAT

#### Abdominal ultrasound orientation

In the early stages of ERAT application, radiological orientation remains to be the mainstay guidance during intubation, stone retrieval, irrigation, and stent deployment. In addition, abdominal ultrasound guidance has also been an important method in ERAT orientation, especially for pregnancy and pediatric appendicitis. Kang *et al.* [21] have reported that abdominal ultrasound orientation is a feasible, safe and effective guidance.

#### Cholangioscopic orientation as an intra-appendiceal application

**Figure 2.**
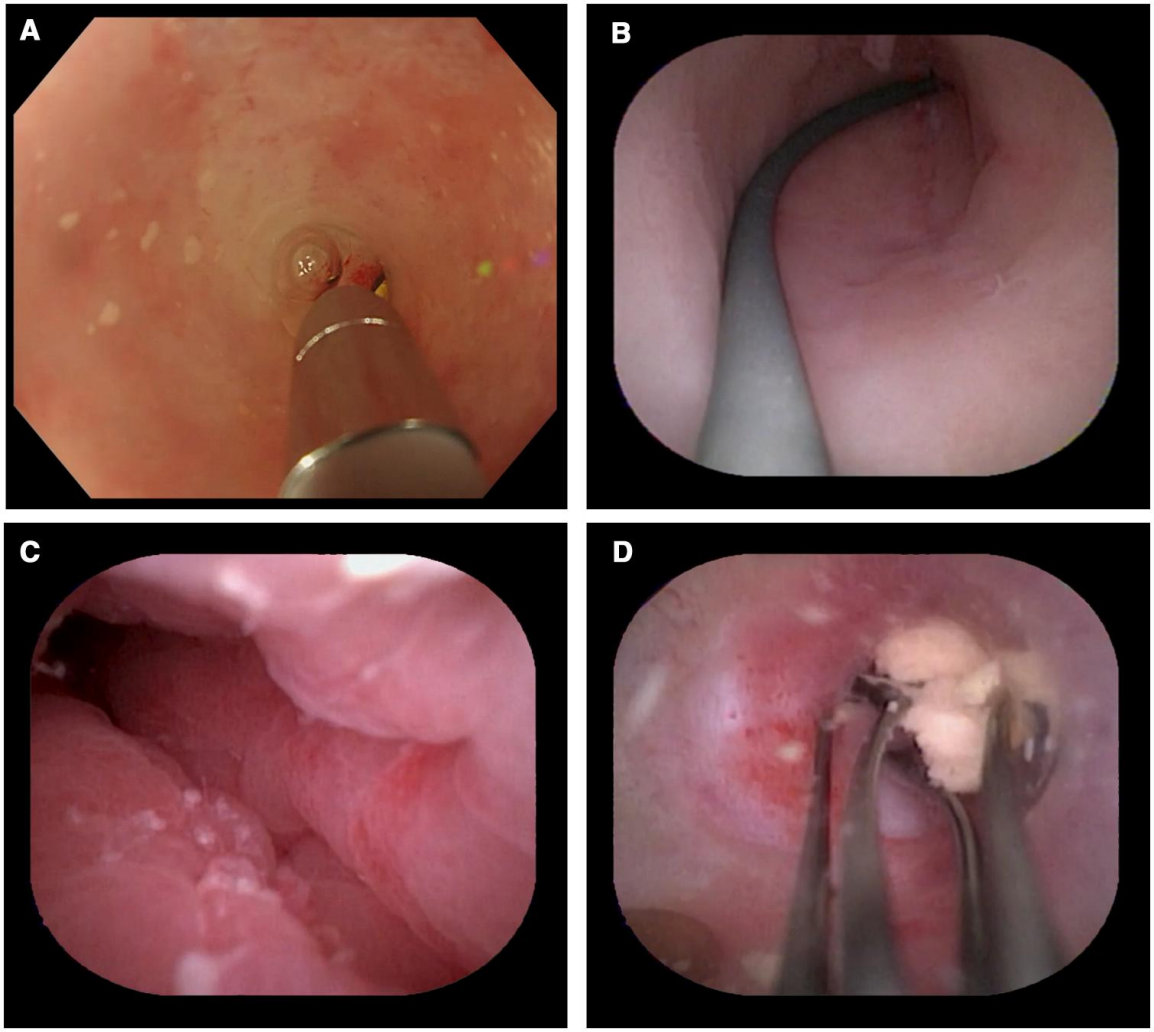
Cholangioscopic orientation during endoscopic retrograde appendicitis therapy. (A) Intubation of the appendix with the cholangioscope. (B) End of the appendix. (C) Observing the condition of the inner wall of the appendix. (D) Fecalith was removed by the basket.

The single-operator cholangioscope has been used in the diagnosis and treatment of biliary system diseases. Kong *et al.* [22] pioneered the introduction of the single-operator cholangioscope to the ERAT procedure, providing a clear and direct view of the appendiceal lumen and mucosa. This approach allows for precise diagnosis of appendiceal disease and facilitating treatments (Figure 2). This orientation method ensures a safe and feasible guidance despite its current higher price and is applicable in selected patients without severe luminal stenosis and angulation.

### Advances in clinical significance

#### Promising to be the gold standard diagnosis of appendicitis

The early diagnosis and classification of acute appendicitis still remain challenging, despite the extensive experience in diagnosis and treatment. The negative appendectomy rate remains high at 8%–15% in clinical practice [23]. A meta-analysis reveals that abdominal ultrasound has a sensitivity of 86% and specificity of 81%, and CT has a sensitivity of 92.3% [1].

Endoscopic Retrograde Appendicography provides a precise and effective diagnostic basis for appendicitis, with a sensitivity and specificity of 92% and 100% [24]. Diagnostic findings of a normal appendix include smooth mucosa around the appendiceal orifice, normal diameter and shape of the appendix, no filling defect, and clear irrigating fluid, which may help avoid unnecessary appendectomy. In acute and chronic appendicitis, the following findings may have diagnostic value: stenosis or dilation of the appendiceal lumen, filling defects (presence of fecalith), non-smooth appendiceal inner wall, and a twisted course of the appendix. Furthermore, direct observation using the peroral cholangioscopy system provides more objective diagnostic evidence, including edema of the appendiceal orifice, intraluminal pus, intraluminal mucosal abnormalities, and the presence of intraluminal fecaliths.

#### Functional gastrointestinal disorders and ERAT

In some endoscopic centers in China, ERAT has been performed on patients with recurrent abdominal pain, bloating, diarrhea, or constipation, along with localized tenderness in the lower right abdomen on physical examination. Some cases are diagnosed with chronic appendicitis, and the referred symptoms are relieved following the ERAT procedure. This result suggests that chronic appendicitis may contribute to functional gastrointestinal diseases and abdominal symptoms, but further evaluation requires additional clinical and basic research.

## Current status of ERAT technology

Between 2009 and December 2023, ERAT has been performed across 33 provinces, municipalities, and special administrative regions in China. Over 150 hospitals have successfully completed over 10,000 ERAT by the end of 2022. More than 100 related articles have been published on ERAT in Chinese, with over 30 ERAT lectures and seminars organized in the recent past two years. A growing number of endoscopists and surgeons are actively engaging in the practice and promotion of ERAT technology.

Bingrong Liu and his team have been devoted to the development and promotion of the ERAT technique both in China and worldwide these years. A number of papers on the ERAT technique and its effectiveness have been published. In 2023, Li *et al.* [25] revealed that ERAT had superiority over antibiotic treatment in terms of pain relief, recurrence reduction, and shorter hospital stay. A meta-analysis by Huang *et al.* [26] indicated the effectiveness of antibiotic treatment for appendicitis but highlighted an increased risk of failure in patients with appendiceal fecalith obstruction. These studies affirm the significant advantages of ERAT, particularly in recurrence prevention, rapid pain relief, and shorter hospital stays. In 2022, a study by Yang *et al.* [27] revealed a one-year curative rate of 92.1% undergoing ERAT, with the ERAT group exhibiting a significantly higher proportion of patients with a visual analog scale score for pain ≤ 3 at 6 hours post-treatment compared with the laparoscopic appendectomy group. The study concluded that ERAT for uncomplicated acute appendicitis is not inferior to laparoscopic appendectomy and boasts unique advantages in terms of non-invasiveness, speed, effectiveness, and safety.

## Upcoming challenges in the new era

ERAT technology has made remarkable strides, with increasing interest both nationally and globally. In spite of many progressions, the clinical application of ERAT is still limited to some experienced centers, almost in China. Otherwise, widespread clinical use of ERAT will provide a more accurate diagnosis of appendiceal disease with the possibility of acquiring biopsies and direct observation using a cholangioscope. Moreover, advances in exclusive instruments, techniques, and training are also essential to improve the wide acceptability and outcomes of ERAT. However, the current landscape of ERAT research predominantly comprises single-center retrospective studies with small sample sizes, short follow-up, and incomplete data collection. Addressing these limitations necessitates comprehensive large-scale prospective randomized controlled studies and long-term follow-up studies. Simultaneously, the ongoing promotion and widespread adoption of ERAT technology present formidable challenges requiring concerted efforts.

Key points for ERAT improvement include as follows:

- **ERAT training:** Though ERAT is not a complex procedure, it demands some skills, especially for intubation and treatments. Endoscopists should be able to recognize the inflamed characteristics of the appendix and the significance of various morphology on radiology and treatment choices. Organizing various training sessions and academic exchange meetings will intensify efforts in promoting technology. Initially, the endoscopist should observe the procedure performed by experienced operators and familiarize themselves with all equipment needed for the intervention including the procedural-related skills.
- **Standards of practice:** Formulating comprehensive standards of practice, to improve ERAT technology and ensure its healthy development.
- **Promote ERAT-related research and development:** Close collaboration with medical technology companies, accelerating the research and application of new instruments in order to improve the worldwide clinical application.
- **Popular propaganda of ERAT technology:** Intensify the public knowledge of appendiceal physiology, and publicize the advantages of ERAT technology and its benefits.
- **Promote the international application of ERAT:** Enhancing international exchanges and promotion through strategies such as publishing SCI articles, international conference lectures, international cooperation, and establishing international ERAT technology training bases. These may promote the international application of ERAT.

## Conclusions

Currently, ERAT is gradually being accepted by more and more doctors and patients as the preferred treatment for acute uncomplicated appendicitis in China. As these challenges mentioned above are addressed, the global adoption and application of ERAT will be imminent. The majority of patients with appendicitis may no longer require appendectomy, and most ERAT can be performed in outpatient or daycare settings. The application of ultra-fine cholangioscope in ERAT allows endoscopists to extend the visual field into the appendiceal cavity, improving the accuracy of diagnosis and treatment of appendicitis, appendiceal polyps, and tumors, which will most likely change the treatment pattern of the entire appendiceal disease.
